# Urine culture and urinalysis utilization practices in United States acute care hospitals between 2017 and 2020

**DOI:** 10.1017/ice.2025.10260

**Published:** 2025-10-24

**Authors:** Nyawung L. Asonganyi, Sophia V. Kazakova, Kelly M. Hatfield, James Baggs, Scott K. Fridkin, Sujan C. Reddy, Joseph Daniel Lutgring

**Affiliations:** 1 Division of Healthcare Quality Promotion, Centers for Disease Control and Prevention, Atlanta, Georgia, USA; 2 Department of Epidemiology, Rollins School of Public Health, Emory University, Atlanta, Georgia, USA; 3 Division of Infectious Diseases, Department of Medicine, Emory University School of Medicine, Atlanta, Georgia, USA; 4 Georgia Emerging Infections Program, Decatur, Georgia, USA

## Abstract

**Objective:**

Inappropriate urine cultures (UCs) are common and lead to inappropriate antimicrobial use. Urinalyses (UAs) have been increasingly incorporated into diagnostic stewardship interventions, but the impact of these interventions nationally has not been assessed. We describe UA and UC utilization practices using a nationwide dataset of patients admitted to acute care hospitals.

**Methods:**

Design, Setting and Participants: We performed a retrospective cohort study of index UCs and their associated UAs performed for adult patients (age ≥ 18 years) admitted in U.S. acute care hospitals, participating in the PINC AI^™^ Healthcare Database (PHD) from January 1, 2017, through December 31, 2020. A positive UA was defined as >10 leukocytes per high power field, positive leukocyte esterase, or positive nitrite.

**Results:**

The overall rate of UCs in this study was 124.7 per 1000 discharges and annual UC rates decreased from 2017 (129.2) to 2020 (120.0). The proportion of UCs that had a positive UA increased from 60.5% in 2017 to 68.1% in 2020; UCs without a UA decreased from 19.3% to 10.5%, and UCs with a negative UA did not significantly change (20.2% to 21.5%). A multivariate multinomial logistic regression model identified male sex, age <65, and a diagnosis of cancer to be predictors of having a UC with a negative UA or no UA.

**Conclusions:**

UC utilization decreased over the study period. The proportion of UCs with a positive UA increased. This may suggest a positive impact of diagnostic stewardship practices at the national level although further progress is needed.

## Introduction

Urinary tract infections (UTIs) are common among hospitalized patients.^1,2^ Approximately 20% of all hospitalized patients receiving antimicrobial therapy are being treated for a UTI.^3^ The diagnosis of UTI is principally based on signs and symptoms, but a urinalysis (UA) can assist with the diagnosis.^4–6^ It may detect either pyuria (presence of leukocytes or leukocyte esterase) or bacteriuria (presence of bacteria or nitrite). One meta-analysis found that presence of leukocyte esterase or nitrite had a likelihood ratio of 4.2 and the absence of both had a likelihood ratio of 0.3 for UTI.^4,7^ A urine culture (UC) can further assist with guiding antimicrobial therapy by identifying the causative organism and providing antimicrobial susceptibility testing results.^8^ In general, patients with asymptomatic bacteriuria should not be treated, except pregnant patients and those undergoing invasive urologic procedures.^9,10^ There is insufficient evidence to recommend for or against treatment of asymptomatic bacteriuria in those with a renal transplant in the past month or those with neutropenia.^9^ Despite recommendations to avoid treatment of asymptomatic bacteriuria, the practice is common.^11,12^

Several diagnostic stewardship initiatives have been demonstrated to reduce the inappropriate use of antimicrobials for hospitalized patients with asymptomatic bacteriuria.^13^ These include requiring documentation of signs or symptoms of UTI prior to ordering UCs, performing conditional reflex UCs (i.e., ordered UCs are only performed by the laboratory if a UA is “positive”), removing UCs from electronic health record order sets when testing is inappropriate, and modifying the way positive UCs are reported.^13–17^ Whether these diagnostic stewardship interventions have been widely adopted and affected nationwide UA and UC test utilization trends is unknown. The practice of ordering conditional reflex UCs has been debated. Still, at least some facilities have adopted it.^13,15,18^ If this practice were adopted, the proportion of UCs with a positive UA would increase, and the proportion of UCs without a UA or with a negative UA would decrease.

We sought to describe UA and UC utilization practices and trends in the United States using a nationwide dataset of patients admitted to acute care hospitals between 2017 and 2020. We categorized patients with a UC into three groups: those with a positive UA, those with a negative UA, and those without a UA. There is no consensus definition of a positive UA, so we chose the most sensitive definition (>10 leukocytes per high power field, presence of leukocyte esterase, or presence of nitrite).^13^ We then determined the proportion of hospitalizations in each of these groups over time, described facility-level variability in the proportions, and investigated patient- and facility-level factors predicting an individual patient’s UC category. We did this to identify which types of facilities and patients may benefit most from diagnostic stewardship efforts.

## Methods

### Study design, patient population, and data source

We performed a retrospective cohort study of index UCs and their associated UAs performed for adult patients (age ≥ 18 years) admitted to a dynamic cohort of U.S. acute care hospitals, participating in the PINC AI^™^ Healthcare Database (PHD) for at least one month from January 1, 2017, through December 31, 2020. The PHD includes all-payer facility- and service-level information on patient encounters, including hospitalizations, patient demographics, billing records, laboratory results, clinical diagnoses, and hospital characteristics.^19^

### Urine culture identification and selection

**Figure 1.**
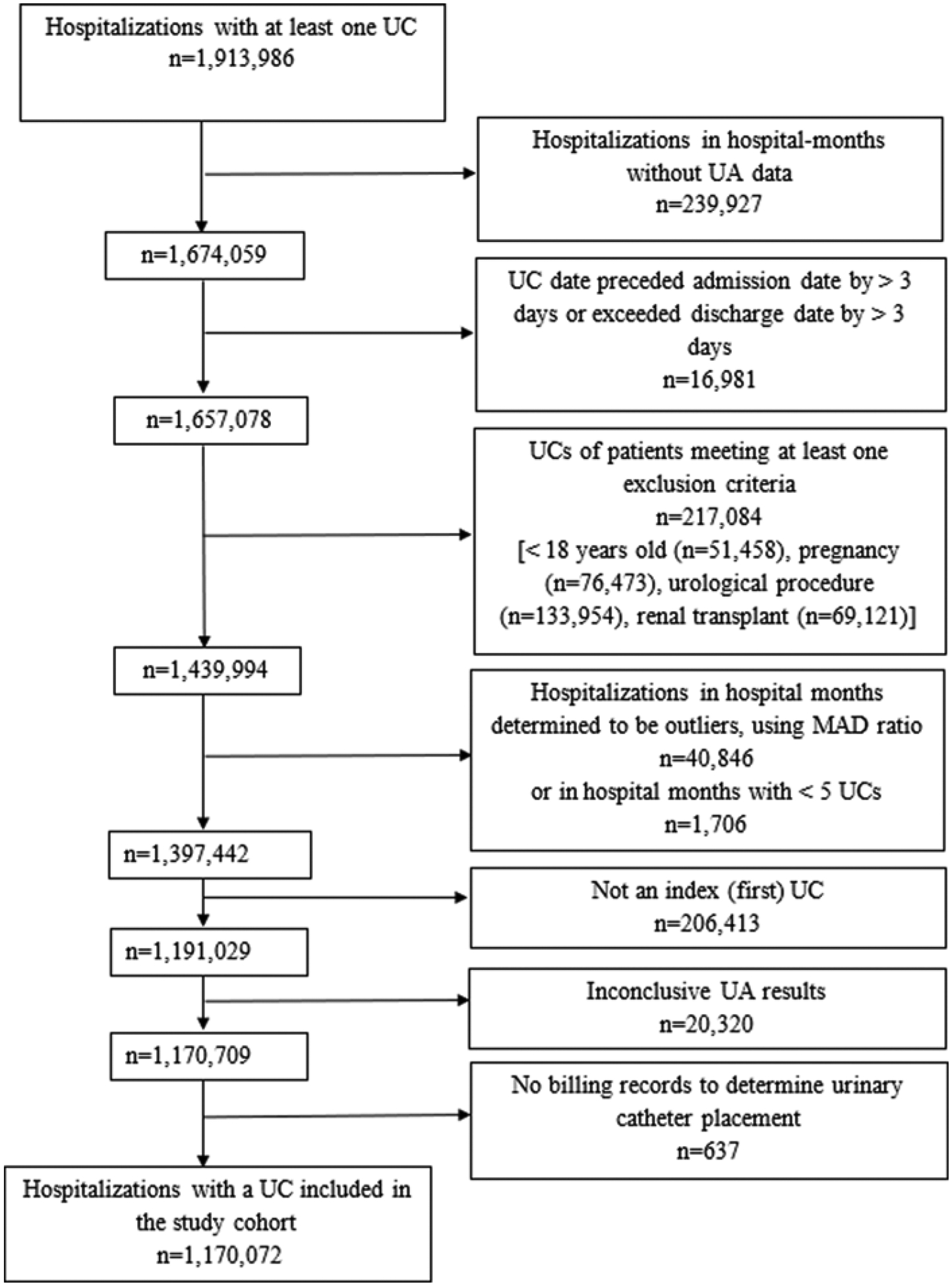
Hospitalizations in U.S. Acute Care Hospitals (*n* = 277) in 2017–2020 with a Urine Culture Included in the Study Cohort, PINC AI^™^ Healthcare Database. *Note*: UC, urine culture; UA, urinalysis; MAD, median absolute deviation.

The process of UC identification and selection is outlined in Figure 1. We identified all UCs associated with an inpatient hospitalization in the PHD by date of specimen collection that were reported in the general and microbiology laboratory data tables (*n* = 1,913,966). In the PHD, UA data is in the general laboratory data and UC data is in the microbiology laboratory data. We excluded hospitalizations that were collected in hospital months that did not submit both UC and UA data (*n* = 239,927) or had a specimen collection date that preceded the patient admission date by >3 days or exceeded the patient discharge date by >3 days (*n* = 16,981). We then excluded hospitalizations with a UC collected from patients <18 years old, pregnant patients, those undergoing a urological procedure, and renal transplant patients, as identified using ICD-10-CM (the International Classification of Diseases, Tenth Revision, Clinical Modification) diagnosis codes (*n* = 217,084) (Supplementary Table S1); pregnant patients, those undergoing a urological procedure, and renal transplant patients may have an indication for the treatment of asymptomatic bacteriuria, hence their exclusion. To ensure the completeness of data reported to PHD and to remove facilities with incomplete reporting, an outlier analysis was conducted using the median absolute deviation (MAD) ratio for monthly UC counts.^20^ If a hospital-month had a UC count associated with a MAD ratio greater than five, all monthly UC and discharge counts for that hospital were reviewed to identify any abnormal patterns. For instance, if a discharge count for a particular month was within the expected range, but the UC count deviated significantly as indicated by the MAD ratio, that month was excluded from analysis. This process resulted in the exclusion of 40,846 hospitalizations. The outlier analysis for UC counts addresses overall issues in laboratory data reporting that could similarly affect UA data and ensures overall data completeness. Additionally, hospitalizations during hospital months with < 5 UCs were excluded (*n* = 1706). Since this study focused on first UC in the hospitalization (i.e., index UC), all subsequent cultures were removed, resulting in the exclusion of 206,413 cultures. Furthermore, UCs with inconclusive UA results (*n* = 20,320), or UCs lacking billing records necessary to determine urinary catheter placement (*n* = 637) were also excluded from the analysis (Figure 1).

### Urine culture rates

In our study, we estimated annual UC rates as the number of discharges with an included UC per 1000 discharges. Discharges were calculated by summing the total number of distinct discharges in each included hospital month by year. We used a negative binomial regression model to assess if there was an observable annual trend in the UC per 1000 discharges.

### Evaluation of urinalysis

UA tests performed within 24 hours before or after UC were identified from general laboratory data using LOINC^®^ codes (Supplementary Table S2). UA tests included leukocyte count, leukocyte esterase, and nitrite. A positive UA was defined as a leukocyte count ≥10 cells per high power field (HPF) OR presence of leukocyte esterase OR presence of nitrite. A UA was categorized as negative if at least one of the UA tests results was reported and interpretable and all reported results were negative (i.e., leukocyte count <10 cells per HPF, absence of leukocyte esterase, and/or absence of nitrite). The UC was considered inconclusive and excluded from the study if all UA tests were uninterpretable or missing. Examples of uninterpretable results included “insufficient quant,” “field obscured by RBC,” and “sample too bloody to perform test.” Based on this UA result evaluation, UCs were categorized into three groups: UC with a positive UA, UC with a negative UA, and UC without a UA.

### Patient and hospital characteristics

For each included UC, we identified relevant patient characteristics from PHD, including sex, age, race, Hispanic ethnicity, hospitalization length of stay, payer, admission type (i.e., emergency, urgent, elective, trauma), admission source, and inpatient death or discharge to hospice. Timing of the UC was categorized as performed within three days of admission or on day four or later of the hospitalization. ICD-10-CM diagnosis codes were used to identify diagnoses during the associated inpatient hospitalization of cancer, diabetes, or UTI (Supplementary Table S3). To examine whether urinary catheter use was associated with any of the three UC categories, we used hospital billing records to identify a charge for a urinary catheter placement during hospitalization (Supplementary Table S4). Using the UC data and the urinary catheter charge date, we grouped urinary catheter placements into four categories: urinary catheter placed on the day of UC, urinary catheter placed 1–10 days prior to the UC, urinary catheter placed >10 days prior to UC, and urinary catheter placed any time after UC. The timing of urinary catheter removal could not be determined. For each UC, relevant hospital characteristics, including teaching status, urban or rural location, number of beds, and region, were also identified from PHD. PHD identifies an urban area as a territory with core census blocks of at least 1000 people per square mile and surrounding census blocks with at least 500 people per square mile; rural areas are those areas not defined as urban.^19^

### Statistical analysis

The three UC categories were stratified by the patient and hospital characteristics above. For facility-level analysis of UC proportions, a monthly count of UCs was obtained for each hospital and the proportion of hospitalizations in each of the three UC categories was calculated. Facility-level variability in the median monthly proportions was described by the median and 25^th^:75^th^ percentiles (Q1:Q3). We used simple logistic regression models to assess the unadjusted odds in the annual proportion of UCs with a positive UA by year from 2017 to 2020.

We performed a multinomial logistic regression analysis to quantify the odds of having a UC with a negative UA or no UA compared to a positive UA for patient and hospitalization characteristics. The covariates assessed in the model were year (continuous), quarter, sex, age (< 65 vs ≥ 65 years old), timing of UC (performed within 3 days of admission or on day four or later of the hospitalization), payor (categorized as Medicare, Medicaid, other), cancer, diabetes, urine catheter placement in ten days before UC, region, urban/rural status, hospital teaching status, and hospital bed size. The model was checked for multicollinearity by fitting a linear regression and assessing tolerance and variance inflation factor. The model accounted for facility-level clustering using generalized estimating equations method. All data were analyzed using SAS 9.4 (SAS Institute, Cary, NC).

## Results

From 2017 to 2020, a total of 1,170,072 UCs, representing 61.1% of all observed UCs, from 277 hospitals met the inclusion criteria for our analysis (Figure 1). The overall UC rate was 124.7 per 1000 discharges; annual rates were decreasing from 2017 to 2020 (129.2, 128.7, 121.7, and 120.0 per 1000 discharges, respectively, test for trend *p* < 0.0001).

Of the included UCs, 760,622 (65.0%) had a positive UA, 246,471 (21.1%) had a negative UA, and 162,979 (13.9%) had no UA (Table 1). During the study period, the proportion of UCs with a positive UA increased from 60.5% in 2017 to 68.1% in 2020. A simple logistic regression model identified a 10% increase in the odds of a UC having a positive UA annually (OR 1.10, *p* < 0.0001). Of the 760,622 UCs with a positive UA, 25,149 (3.3%) were only considered positive because of the presence of nitrite. Meanwhile, UCs without a UA decreased from 19.3% to 10.5%, and the proportion of UCs with a negative UA remained relatively stable, ranging from 20.2% to 21.5% across the four years (Table 1).

Most UCs were obtained from female patients (60.8%), with 70.6% of UCs from females showing a positive UA, compared to 56.3% of UCs from males. A diagnosis of UTI was present in 43.6% of the included hospitalizations, with UTI being more common in the positive UA group (56.6%) and less frequent among UCs with a negative UA (10.2%). A diagnosis of cancer was noted in 22.2% of hospitalizations, slightly more frequent in the negative UA group (25.1%) and no UA group (24.4%) compared to the positive UA group (20.8%). Diabetes was present in 38.3% of hospitalizations, with no notable differences across the UC groups.

Based on billing data, urinary catheter placement was documented in 14.4% of included hospitalizations. Catheter placement within 1–10 days prior to the UC was more common in the UC group without a UA (5.1%) compared to the UC with a positive or negative UA groups (2.8% for both). Medicare was the most common payor (68.0%), followed by Medicaid (11.4%) and Managed Care (10.9%). UCs with a positive UA were more frequent in Medicare hospitalizations (70.8%) compared to those with negative UAs (65.5%) or no UA (60.8%). Most UCs (89.5%) were performed on or before day 3 of admission; however, UCs obtained after the 3^rd^ day of hospitalization were more common in the no UA group (18.4%) compared to the positive UA group (8.9%).

The facility variability in median proportions of UCs in each UC group was as follows: the positive UA group had a median proportion of 75.0% (interquartile range (Q1:Q3): 55.6%–86.4%), the negative UA group median proportion was 15.0% (6.5%–28.6%), and the no UA group’s median proportion was 5.3% (2.1%–10.5%) (Supplementary Figure S1). A similar breakdown of the variability in median proportions based on hospital characteristics is provided in Supplementary Table S5.

Results from multivariate analysis estimating the odds of having a negative or no UA for various patient characteristics are summarized in Table 2. The odds of a UC without UA decreased by 22% annually (OR 0.78; 95% CI, 0.69, 0.87). Significant seasonal variation was observed, with January-February and March-May showing 10% and 23% greater odds, respectively, of a negative UA or no UA compared to September–November. Male patients had 2.10 times higher odds of having a negative UA (95% CI, 2.03, 2.18) and 1.41 times higher odds of having no UA (95% CI: 1.38, 1.51) compared to female patients. Patients under 65 years had 41% higher odds of a negative UA (OR 1.41; 1.35, 1.47) and 20% higher odds of no UA (OR 1.20; 1.09, 1.33) compared to those aged 65 and older. UCs collected on or after day 4 of hospitalization were associated with 2.26 times higher odds of having no UA compared to those collected within the first 3 days. Compared to patients with Medicare, those with Medicaid or other non-Medicare/non-Medicaid coverage had 10% (OR 1.10; 95% CI: 1.06, 1.16) and 25% (OR 1.25; 95% CI: 1.18, 1.32) higher odds of having a negative UA, respectively. Cancer diagnosis was linked to 20% higher odds of a negative UA (OR 1.20; 95% CI: 1.13, 1.27) higher odds of having a negative UA and 16% higher odds of having no UA (OR 1.16; 95% CI: 1.05, 1.28) while diabetes was associated with lower odds of a negative UA (OR 0.93; 0.90, 0.95). Geographically, UCs from hospitals in the Midwest had 66% lower odds of no UA (OR 0.34; 95% CI: 0.19, 0.62), while UCs from rural hospitals had 36% higher odds of having a negative UA (OR 1.36; 95% CI: 1.03, 1.80).

## Discussion

This large nationwide study of acute care hospitalizations with index UC revealed notable trends between 2017 and 2020. Despite an overall decrease in the rate of UCs, there was an increase in the proportion of UCs accompanied by a positive UA, rising from 60.5% in 2017 to 68.1% in 2020. At the same time, the proportion of UCs without a UA decreased from 19.3% to 10.5%. Possible explanations for these findings include an increase in the number of facilities performing conditional reflex UCs, other diagnostic stewardship initiatives, changes in clinician test ordering practices for other reasons, or changes in the epidemiology of UTIs.

A multivariate multinomial logistic regression model identified male sex, age <65, and a diagnosis of cancer as predictors of having a UC with either a negative UA or no UA. These findings may suggest where UC stewardship practices remain suboptimal.^13^ In addition, patients with Medicaid had higher odds of having a UC with a negative UA. Others have found payor type to be associated with adverse health outcomes.^21^ Also, facilities in the Midwest were less likely to have a UC without a UA. It is known that both infections and culturing practices can vary by region.^22,23^

There is limited national data examining the relationship between UCs and UAs. Horstman et al. evaluated urine testing patterns from 2009 to 2014 using a national dataset and found that both UC and UA tests were common; however, they did not stratify their UC data by UA result.^24^ Similarly, a single-center study conducted between 2008 and 2013 found that 20.2% of UCs were performed without a UA, a proportion comparable to our finding in 2017.^25^

UC stewardship interventions aim to optimize the diagnosis of UTIs and to prevent the unnecessary treatment of asymptomatic bacteriuria.^13^ Strategies demonstrating an increase in appropriate antimicrobial use or a reduction in inappropriate UC ordering include using clinical decision support to optimize UC ordering, having a conditional reflex UC policy, and providing nursing education.^18,26,27^ Adoption of these UC practices may be responsible for this study’s observed findings of decreased UC rate and the concurrent increase in the proportion of UCs with a positive UA.

There are several limitations of our study. First, we utilized an administrative dataset, and we were unable to determine the appropriateness of the UCs and UAs included. The secondary use of this dataset may also cause misclassification of the study outcomes and exposures. Additionally, the hospitals included in the study represent a convenience sample, limiting the generalizability of our findings to hospitals across the United States. We also did not analyze the specific results of the UCs or potential impacts on antimicrobial use. Future work could examine these impacts. Lastly, information regarding diagnostic stewardship practices at the participating facilities such as the use of conditional reflex UCs was not available. This prevents us from firmly knowing the reasons for the changing UC ordering practices.

In conclusion, UCs are a significant contributor to inappropriate antimicrobial use in hospitals and are a key focus of the Society for Healthcare Epidemiology of America Diagnostic Stewardship Taskforce.^28^ This study indicates some improvement in UC utilization between 2017 and 2020. As diagnostic stewardship interventions become more widely implemented, it will be critical for future studies to evaluate their impact on clinical practices and patient outcomes.

## Supplementary Material

Supplement

**Supplementary material.** To view supplementary material for this article, please visit https://doi.org/10.1017/ice.2025.10260.

## Figures and Tables

**Table 1.** Characteristics of hospitalizations with an index urine culture performed at U.S. acute care hospitals (*n* = 277), 2017–2020

Characteristics^a^	Urine culture with positive urinalysis	Urine culture with negative urinalysis	Urine culture without urinalysis	All urine cultures
Total urine cultures, n (row %)	760,622 (65.0)	246,471 (21.1)	162,979 (13.9)	1,170,072 (100)
Year, n (row %)^b^				
2017	159,258 (60.5)	53,221 (20.2)	50,696 (19.3)	263,175 (100)
2018	206,723 (65.4)	66,669 (21.1)	42,654 (13.5)	316,046 (100)
2019	204,168 (65.6)	66,886 (21.5)	40,260 (12.9)	311,314 (100)
2020	190,473 (68.1)	59,695 (21.4)	29,369 (10.5)	279,537 (100)
Quarter				
1 (Dec, Jan, Feb)	182,801 (24.0)	63,136 (25.6)	43,126 (26.5)	289,063 (24.7)
2 (March, April, May)	183,951 (24.2)	60,001 (24.3)	40,662 (24.9))	284,614 (24.3)
3 (June, July, Aug)	197,714 (26.0)	60,603 (24.6)	40,431 (24.8)	298,748 (25.5)
4 (Sept, Oct, Nov)	196,156 (25.8)	62,731 (25.5)	38,760 (23.8)	297,647 (25.4)
Patient characteristics				
Sex^c^				
Female	502,657 (66.1)	116,682 (47.3)	92,626 (56.8)	711,965 (60.8)
Male	257,962 (33.9)	129,787 (52.7)	70,348 (43.2)	458,097 (39.2)
Age (in years)				
18–54	145,127 (19.1)	63,936 (25.9)	37,177 (22.8)	246,240 (21.0)
55–64	118,427 (15.6)	48,172 (19.5)	29,778 (18.3)	196,377 (16.8)
65–74	170,768 (22.5)	55,748 (22.6)	38,162 (23.4)	264,678 (22.6)
75+	326,300 (42.9)	78,615 (31.9)	57,862 (35.5)	462,777 (39.6)
Race				
White	593,712 (78.1)	185,733 (75.4)	116,318 (71.4)	895,763 (76.6)
Black	100,597 (13.2)	34,603 (14.0)	30,361 (18.6)	165,561 (14.1)
Asian	13,858 (1.8)	4,628 (1.9)	3,594 (2.2)	22,080 (1.9)
Other	40,315 (5.3)	17,428 (7.1)	9,882 (6.1)	67,625 (5.8)
Unknown	12,140 (1.6)	4,079 (1.7)	2,824 (1.7)	19,043 (1.6)
Hispanic ethnicity				
Yes	46,551 (6.1)	12,701 (5.2)	7,372 (4.5)	66,624 (5.7)
No	604,225 (79.4)	174,609 (70.8)	133,223 (81.7)	912,057 (77.9)
Unknown	109,846 (14.4)	59,161 (24.0)	22,384 (13.7)	191,391 (16.4)
Inpatient death or discharge to hospice	78,209 (10.3)	23,337 (9.5)	17,794 (10.9)	119,340 (10.2)
Inpatient diagnoses				
Urinary tract infection	430,202 (56.6)	25,239 (10.2)	54,926 (33.7)	510,367 (43.6)
Cancer	158,521 (20.8)	61,880 (25.1)	39,723 (24.4)	260,124 (22.2)
Diabetes	295,813 (38.9)	90,022 (36.5)	62,695 (38.5)	448,530 (38.3)
Urinary catheter^d^				
No urinary catheter placed	654,980 (86.1)	210,076 (85.2)	136,824 (84.0)	1,001,880 (85.6)
Urinary catheter placed during hospitalization	105,642 (13.9)	36,395 (14.8)	26,155 (16.0)	168,192 (14.4)
Placed on the day of urine culture	56,828 (7.5)	21,008 (8.5)	11,858 (7.3)	89,694 (7.7)
Placed 1–10 days before urine culture	21,142 (2.8)	6,927 (2.8)	8,271 (5.1)	36,340 (3.1)
Placed >10 days before urine culture	1,583 (0.2)	382 (0.2)	789 (0.5)	2,754 (0.2)
Placed after urine culture	26,089 (3.4)	8,078 (3.3)	5,237 (3.2)	39,404 (3.4)
Length of stay (days), median (Q1:Q3)	5 (3–8)	5 (3–8)	5 (3–9)	5 (3–8)
Admission source				
Non-healthcare facility	639,597 (84.1)	206,077 (83.6)	134,567 (82.6)	980,241 (83.8)
Clinic	31,959 (4.2)	13,076 (5.3)	6,791 (4.2)	51,826 (4.4)
Transfer from skilled nursing facility or intermediate care facility	28,934 (3.8)	5,566 (2.3)	4,211 (2.6)	38,711 (3.3)
Transfer from health facility	55,951 (7.4)	20,202 (8.2)	15,452 (10.1)	92,605 (7.9)
Court or law enforcement	499 (0.1)	259 (0.1)	134 (0.1)	892 (0.1)
Transfer from unit in the same hospital	1,450 (0.2)	352 (0.1)	404 (0.2)	2,206 (0.2)
Transfer from ambulatory surgery center	576 (0.1)	515 (0.2)	214 (0.1)	1,305 (0.1)
Transfer from hospice	131 (0.0)	34 (0.0)	27 (0.0)	192 (0.0)
Information not available	1,525 (0.2)	390 (0.2)	179 (0.1)	2,094 (0.2)
Admission type				
Emergency	657,649 (86.5)	197,413 (80.1)	123,558 (75.8)	978,620 (83.6)
Urgent	61,432 (8.1)	28,654 (11.6)	25,046 (15.4)	115,132 (9.8)
Elective	31,715 (4.2)	15,343 (6.2)	12,545 (7.7)	59,603 (5.1)
Trauma center	6,578 (0.9)	4,203 (1.7)	1,252 (0.8)	12,033 (1.0)
Information not available	3,248 (0.4)	858 (0.3)	578 (0.4)	4,684 (0.4)
Payor category				
Medicare	538,805 (70.8)	149,902 (60.8)	106,736 (65.5)	795,443 (68.0)
Medicaid	79,508 (10.5)	33,595 (13.6)	20,635 (12.7)	133,738 (11.4)
Managed care	78,495 (10.3)	34,568 (14.0)	15,053 (9.2)	128,116 (10.9)
Commercial, workers’ compensation, employer contract	44,659 (5.9)	15,651 (6.4)	9,509 (5.8)	69,819 (6.0)
Charity, indigent, self-pay, other government payor	19,155 (2.5)	12,755 (5.2)	11,046 (6.8)	42,956 (3.7)
Specimen day of collection				
< day 4	692,613 (91.1)	221,657 (89.9)	132,959 (81.6)	1,047,229 (89.5)
≥ day 4	68,009 (8.9)	24,814 (10.1)	30,020 (18.4)	122,843 (10.5)
Hospital characteristics^e^				
Geographic location				
Urban	629,435 (82.8)	192,444 (78.1)	134,129 (82.3)	956,008 (81.7)
Rural	131,187 (17.2)	54,027 (21.9)	28,850 (17.7)	214,064 (18.3)
Teaching hospital				
Yes	343,204 (45.1)	123,046 (49.9)	71,189 (43.7)	537,439 (45.9)
No	417,418 (54.9)	123,425 (50.1)	91,790 (56.3)	632,633 (54.1)
Number of beds				
< 100	55,026 (7.2)	13,533 (5.5)	5,881 (3.6)	74,440 (6.4)
100–199	111,727 (14.7)	31,448 (12.8)	23,030 (14.1)	166,205 (14.2)
200–299	106,850 (14.0)	45,945 (18.6)	35,694 (21.9)	188,489 (16.1)
300–399	144,522 (19.0)	49,778 (20.2)	26,582 (16.3)	220,882 (18.9)
400–499	69,648 (9.2)	23,727 (9.6)	17,881 (11.0)	111,256 (9.5)
> 499	272,849 (35.9)	82,040 (33.3)	53,911 (33.1)	408,800 (34.9)
Facility region				
South	484,685 (63.7)	132,792 (53.9)	112,858 (69.2)	730,335 (62.4)
Midwest	145,880 (19.2)	45,824 (18.6)	17,489 (10.7)	209,193 (17.9)
Northeast	106,387 (14.0)	58,683 (23.8)	24,561 (15.1)	189,631 (16.2)
West	23,670 (3.1)	9,172 (3.7)	8,071 (5.0)	40,913 (3.5)

a Characteristics are described by column percentages (unless noted otherwise).

b Simple logistic regression models identified a significant 10% increase in the odds of a urine culture having a positive urinalysis annually (OR = 1.10, *p* < 0.001).

c Ten patients have missing sex information.

d Urinary catheter placement during hospitalization was determined using hospital billing records and categorized by date of placement in relationship to the urine culture date.

e Hospital characteristics are described by hospitalization

* Data source was a dynamic cohort of U.S. acute care hospitals, participating in the PINC AI^™^ Healthcare Database (PHD).

**Table 2.** Multivariate multinomial logistic regression model investigating predictors of a urine culture with negative urinalysis and urine culture without urinalysis in acute care hospitals (*N* = 277), 2017–2020^a^

	Urine culture with negative urinalysis		Urine culture without urinalysis	
Characteristics	ROR (95% CI)	*P*-Value	ROR (95% CI)	*P*-Value
Year	0.97 (0.91, 1.03)	0.3399	0.78 (0.69, 0.87)	< 0.0001
1 (Dec, Jan, Feb)	1.11 (1.07, 1.15)	< 0.0001	1.14 (1.06, 1.23)	0.0007
2 (March, April, May)	1.10 (1.05, 1.15)	< 0.0001	1.23 (1.11, 1.36)	< 0.0001
3 (June, July, Aug)	1.00 (0.96, 1.03)	0.8123	1.04 (0.96, 1.14)	0.3195
4 (Sept, Oct, Nov) (Referent)				
Patient Characteristics				
Sex				
Female (Referent)				
Male	2.10 (2.03, 2.18)	< 0.0001	1.44 (1.38, 1.51)	< 0.0001
Age (in years)				
< 65	1.41 (1.35, 1.47)	< 0.0001	1.20 (1.09, 1.33)	0.0002
≥ 65 (Referent)				
Race				
White (Referent)				
Black	1.10 (0.97, 1.25)	0.1287	1.44 (0.99, 2.11)	0.0579
Asian	1.05 (0.77, 1.43)	0.7428	1.05 (0.74, 1.48)	0.7955
Other	1.21 (0.96, 1.52)	0.1074	1.02 (0.71, 1.47)	0.9039
Unknown	1.10 (0.92, 1.31)	0.3055	1.16 (0.82, 1.63)	0.4019
Cancer				
Yes	1.20 (1.13, 1.27)	< 0.0001	1.16 (1.05, 1.28)	0.0026
No (Referent)				
Diabetes				
Yes	0.93 (0.90, 0.95)	< 0.0001	0.97 (0.93, 1.01)	0.0899
No (Referent)				
Urinary catheter				
No urinary catheter placed, placed on the day of UC, placed >10 days before UC, or placed after UC (Referent)				
Placed 1–10 days before urine culture	0.93 (0.79, 1.10)	0.4047	1.23 (1.00, 1.52)	0.0551
Payor category				
Medicare (Referent)				
Medicaid	1.10 (1.06, 1.16)	< 0.0001	1.08 (0.99, 1.18)	0.1018
Other	1.25 (1.18, 1.32)	< 0.0001	1.07 (0.99, 1.17)	0.0863
Day of urine culture				
< 4 days of hospitalization (Referent)				
≥ 4 days of hospitalization	1.09 (0.99, 1.20)	0.0927	2.26 (1.92, 2.66)	< 0.0001
Hospital characteristics				
Geographic location				
Urban (Referent)				
Rural	1.36 (1.03, 1.80)	0.0327	1.09 (0.62, 1.92)	0.7729
Teaching hospital				
Yes	0.97 (0.69, 1.37)	0.8602	1.09 (0.46, 2.58)	0.8479
No (Referent)				
Number of beds				
< 200	0.90 (0.60, 1.35)	0.6189	0.87 (0.37, 2.07)	0.7599
200–399	1.24 (0.85, 1.79)	0.2594	1.19 (0.53, 2.67)	0.6806
> 399 (Referent)				
Facility region				
South	0.77 (0.41, 1.44)	0.4082	0.65 (0.37, 1.15)	0.1399
Midwest	0.94 (0.47, 1.85)	0.8508	0.34 (0.19, 0.62)	0.0004
Northeast	1.46 (0.72, 2.97)	0.2919	0.65 (0.27, 1.54)	0.3257
West (Referent)				

Abbreviations: UC, urine culture; ROR, relative odds ratio; 95% CI, 95% Confidence Interval.

a The comparison category is a urine culture with positive urinalysis. All characteristics listed in the table were included in the multinomial logistic regression, which adjusted for clustering of observations within hospitals.

* Data source was a dynamic cohort of U.S. acute care hospitals, participating in the PINC AI^™^ Healthcare Database (PHD).
