# Supplementary material for: Urine culture and urinalysis utilization practices in United States acute care hospitals between 2017 and 2020
Source: Infect Control Hosp Epidemiol. Author manuscript; Available in PMC 2025 Nov 19. (PMC12624373; doi:10.1017/ice.2025.10260)
Supplement: Supplementary file 1 [file NIHMS2122834-supplement-Supplement.docx]

**Supplementary Materials**

**Supplementary Table S1.** ICD-10-CM Codes to Identify Medical Conditions and Procedures to be Excluded from the Study

| **Condition** | **ICD-10-CM Diagnosis Codes** | **ICD-10-CM Procedure Codes** |
| --- | --- | --- |
| Pregnancy | Z33.X, Z34.X, Z36.X - Z38.X, Z3A.X, O10.X – O16.X, O20.X – O48.X, O60.X – O82.X, O85.X – O92.X, O94.X, O98.X, O99.X, O9A.X | 10.X |
| Urological Procedures | Z48.816 | 0T.X, 0V.X |
| Renal Transplant | Z48.22, Z94.0, T86.10, T86.11, T86.12, T86.13, T86.19 |  |

**Supplementary Table S2.** Urinalysis (UA) LOINC® Codes Used to Identify UA Tests in the PINC AI™ Healthcare Database

| **UA Test LOINC® Code** | **Long Common Name** | **Test Type** |
| --- | --- | --- |
| 5821-4 | Leukocytes [#/Area] in Urine Sediment by Microscopy High Power Field | Leukocyte Count |
| 30405-5 | Leukocytes [#/Volume] in Urine | Leukocyte Count |
| 33052-2 | Leukocytes [Presence] in Urine | Leukocyte Count |
| 24122-4 | Leukocytes [#/Volume] in Urine by Manual Count | Leukocyte Count |
| 20408-1 | Leukocytes [#/Volume] in Urine by Test Strip | Leukocyte Count |
| 53316-6 | Leukocytes [Presence] in Urine by Automated | Leukocyte Count |
| 58805-3 | Leukocytes [#/Volume] in Urine by Automated Test Strip | Leukocyte Count |
| 46702-7 | Leukocytes [#/Area] in Urine Sediment by Automated Count | Leukocyte Count |
| 20455-2 | Leukocytes [Presence] in Urine Sediment by Light Microscopy | Leukocyte Count |
| 51487-7 | Leukocytes [#/Volume] in Urine by Automated Count | Leukocyte Count |
| 5799-2 | Leukocyte Esterase [Presence] in Urine by Test Strip | Leukocyte Esterase |
| 27297-1 | Leukocyte Esterase [Units/Volume] in Urine | Leukocyte Esterase |
| 50558-6 | Leukocyte Esterase [Presence] in Urine by Automated Test Strip | Leukocyte Esterase |
| 5802-4 | Nitrite [Presence] in Urine by Test Strip | Nitrite |
| 2657-5 | Nitrite [Mass/Volume] in Urine | Nitrite |
| 60026-2 | Nitrite [Presence] in Urine by Automated Test Strip | Nitrite |

**Supplementary Table S3.** ICD-10-CM Administrative Codes Used to Identify Cancer, Diabetes, and UTI 

| **Condition** | **ICD-10-CM Diagnosis Code** |
| --- | --- |
| Cancer | C00.0 - C00.6, C00.8 - C00.9, C01, C02.0 - C02.4, C02.8 - C02.9, C03.0, C03.1, C03.9, C04.0, C04.1, C04.8, C04.9, C05.0, C05.1, C05.8, C05.9, C06.0, C06.1, C06.2, C06.80, C06.89, C06.9, C07 C08.0, C08.1, C08.9, C09.0, C09.1, C09.8, C09.9, C10.0 - C10.4, C10.8, C10.9, C11.0 - C11.3, C11.8, C11.9, C12, C13.0 - C13.2, C13.8, C13.9, C14.0, C14.2, C14.8, C15.3 - C15.5, C15.8, C15.9, C16.0 - C16.6, C16.8, C16.9, C17.0 - C17.3, C17.8, C17.9, C18.0 - C18.9, C19, C20, C21.0 - C21.2, C21.8, C22.0 - C22.4, C22.7 - C22.9, C23, C24.0, C24.1, C24.8, C24.9, C25.0 - C25.4, C25.7 - C25.9, C26.0, C26.1, C26.9, C30.0, C30.1, C31.0 - C31.3, C31.8, C31.9, C32.0 - C32.3, C32.8, C32.9, C33, C34.00 - C34.02, C34.10 - C34.12, C34.2, C34.30 - C34.32, C34.80 - C34.82, C34.90 - C34.92, C37, C38.0 - C38.4, C38.8, C39.0, C39.9, C40.00 - C40.02, C40.10 - C40.12, C40.20 - C40.22, C40.30 - C40.32, C40.80 - C40.82, C40.90 - C40.92, C41.0 - C41.4, C41.9, C43.0, C43.10, C43.111, C43.112, C43.121, C43.122, C43.20 - C43.22, C43.30, C43.31, C43.39, C43.4, C43.51, C43.52, C43.59, C43.60 - C43.62, C43.70 - C43.72, C43.8, C43.9, C44.00, C44.09, C44.101, C44.1021, C44.1022, C44.1091, C44.1092, C44.191, C44.1921, C44.1992, C44.201, C44.202, C44.209, C44.291, C44.292, C44.299, C44.300, C44.301, C44.309, C44.390, C44.391, C44.399, C44.40, C44.49, C44.500, C44.501, C44.509, C44.590, C44.591, C44.599, C44.601, C44.602, C44.609, C44.691, C44.692, C44.699, C44.701, C44.702, C44.709, C44.791, C44.792, C44.799, C44.80, C44.89, C44.90, C44.99, C45.0 - C45.2, C45.7, C45.9, C46.0 - C46.4, C46.50 - C46.52, C46.7, C46.9, C47.0, C47.10 - C47.12, C47.20 - C47.22, C47.3 - C47.6, C47.8, C47.9, C48.0 - C48.2, C48.8, C49.0, C49.10 - C49.12, C49.20 - C49.22, C49.3 - C49.6, C49.8, C49.9, C49.10 - C49.A5, C49.A9, C4A.10, C4A.111, C4A.112, C4A.121, C4A.122, C4A.20 - C4A.22, C4A.30, C4A.30, C4A.31, C4A.39, C4A.4, C4A.51, C4A.52, C4A.59 - C4A.62, C4A.70 - C4A.72, C4A.8, C4A.9, C50.011, C50.012, C50.019, C50.021, C50.022, C50.029, C50.111, C50.112, C50.119, C50.121, C50.122, C50.129, C50.211, C50.212, C50.219, C50.221, C50.222, C50.229, C50.311, C50.312, C50.119, C50.321, C50.321, C50.322, C50.329, C50.411, C50.412, C50.419, C50.421, C50.422, C50.429, C50.511, C50.512, C50.519, C50.521, C50.522, C50.529, C50.611, C50.612, C50.619,  C50.621, C50.622, C50.629, C50.811, C50.812, C50.819, C50.821, C50.822, C50.829, C50.911, C50.912, C50.919, C50.921, C50.922, C50.929, C51.0 - C51.2, C51.8, C51.9, C52, C53.0, C53.1, C53.8, C53.9, C54.0 - C54.3, C54.8, C54.9, C55, C56.1, C56.2, C56.9, C57.00 - C57.02, C57.10 - C57.12, C57.20 - C57.22, C57.3, C57.4, C57.7 - C57.9, C58, C60.0 - C60.2, C60.8, C60.9, C61, C62.00 - C62.02, C62.10 - C62.12, C62.90 - C62.92, C63.00 - C63.02, C63.10 - C63.12, C63.2, C63.7 - C63.9, C64.1, C64.2, C64.9, C65.1, C65.2, C65.9, C66.1, C66.2, C66.9, C67.0 - C67.9, C68.0, C68.1, C68.8, C68.9, C69.00 - C69.02, C69.10 - C69.12, C69.20 - C69.22, C69.30 - C69.32, C69.40 - C69.42, C69.50 - C69.52, C69.60 - C69.62, C69.80 - C69.82, C69.90 - C69.92, C70.0, C70.1, C70.9, C71.0 - C71.9, C72.0, C72.1, C72.20 - C72.22, C72.30 - C72.32, C72.40 - C72.42, C72.50, C72.59, C72.9, C73, C74.00 - C74.02, C74.10 - C74.12, C74.90 - C74.92, C75.0 - C75.5, C75.8, C75.9, C76.0 - C76.3, C76.40 - C76.42, C76.50 - C76.52, C76.8, C77.0 - C77.5, C77.8, C77.9, C78.00 - C78.02, C78.1, C78.2, C78.30, C78.39, C78.4 - C78.7, C78.80, C78.89, C79.00 - C79.02, C79.10, C79.11, C79.19, C79.2, C79.31, C79.32, C79.40, C79.49, C79.51, C79.52, C79.60 - C79.62, C79.70 - C79.72, C79.81, C79.82, C79.89, C79.9, C7A.00, C7A.010 - C7A.012, C7A.20 - C7A.26, C7A.029, C7A.090 - C7A.096, C7A.098, C7A.1, C7A.012, C7A.8, C7B.00 - C7B.04, C7B.09, C7B.1, C7B.8, C80.0 - C80.2, C80.8, C81.00 - C81.49, C81.70 - C81.79, C81.90 - C81.99, C82.00 - C82.69, C82.80 - C82.99, C83.00 - C83.19, C83.30 - C83.39, C83.50 - C83.59, C83.70 - C83.99, C84.00 - C84.19, C84.40 - C84.49, C84.60 - C84.79, C84.90 - C84.99, C84.A0 - C84.A9, C84.Z0 - C84.Z9, C85.10 - C85.29, C85.80 - C85.99, C86.0 - C86.6, C88.0, C88.4, C88.8, C88.9, C90.00 - C90.02, C90.10 - C90.12, C90.20 - C90.22, C90.30 - C90.32, C91.00 - C91.02, C91.10 - C91.12, C91.30 - C91.32, C91.40 - C91.42, C91.50 - C91.52, C91.60 - C91.62, C91.90 - C91.92, C91.A0 - C91.A2, C91.Z0 - C91.Z2, C92.00 - C92.02, C92.10 - C92.12, C92.20 - C92.22, C92.30 - C92.32, C92.40 - C92.42, C92.50 - C92.52, C92.60 - C92.62, C92.90 - C92.92, C92.A0 - C92.A2, C92.Z0 - C92.Z2, C93.00 - C93.02, C93.10 - C93.12, C93.30 - C93.32, C93.90 - C93.92, C93.Z0 - C93.Z2,  C94.00 - C94.02, C94.20 - C94.22, C94.30 - C94.32, C94.40 - C94.42, C94.6, C94.80 - C94.82, C95.00 - C95.02, C95.10 - C95.12, C95.90 - C95.92, C96.20, C96.21, C96.22, C96.29, C96.4, C96.60, C96.9, C96.A, C96.Z, D37.01, D37.02, D37.030 - D37.032, D37.039, D37.04 - D37.05, D37.09, D37.1 - D37.6, D37.8, D37.9, D38.0 - D38.6, D39.0, D39.10 - D39.12, D39.2, D39.8, D39.9, D40.0, D40.10 - D40.12, D40.8, D40.9, D41.00 - D41.02, D41.10 - D41.12, D41.20 - D41.22, D41.3, D41.4, D41.8, D41.9, D42.0, D42.1, D42.9, D43.0 - D43.4, D43.8, D43.9, D44.0, D44.10 - D44.12, D44.2 - D44.7, D44.9, D45, D46.22, D47.01, D47.02, D47.09, D47.Z9, D47.1, D47.2, D47.4, D47.9, D47.Z1, D47.Z9, D48.0 - D48.5, D48.60 - D48.62, D48.7, D48.9, D49.0 - D49.4, D49.511, D49.512, D49.519, D49.59, D49.6, D49.7, D49.81, D49.89, D49.9, D61.82, D69.51, D69.59, D69.6, D75.81, D70.3, D70.8, D70.9, D72.810, D72.818, D72.819, D72.9, D73.0 - D73.5, D73.81, D73.89, D73.9, D73.9, D75.89, D75.9, D89.0 - D89.3, D89.40 - D89.43, D89.39, D89.810 - D89.813, D89.82, D89.89, Q85.00 - Q85.03, Q85.09, R64, R76.8, R76.9, S36.00XA, S36.020A, S36.021A, S36.029A - S36.032A, S36.039A, S36.09XA |
| Diabetes | E08.00, E08.01, E08.10, E08.11, E08.21, E08.22, E08.29, E08.311, E08.319, E08.3211 - E08.3213, E08.3219, E08.3291 - E08.3293, E08.3299, E08.3411 - E08.3413, E08.3419, E08.3491 - E08.3493, E08.3499, E08.3511 - E08.3513, E08.3519, E08.3521 - E08.3523, E08.3529, E08.3531 - E08.3533, E08.3539, E08.3591 - E08.3593, E08.3599, E08.36, E08.37X1 - E08.37X3, E08.37X9, E08.39 - E08.39 - E08.44, E08.49, E08.51, E08.52, E08.59, E08.610, E08.618, E08.620 - E08.622, E08.628, E08.630, E08.638, E08.641, E08.649, E08.65, E08.69, E08.8, E08.9, E09.00, E09.01, E09.10, E09.11, E09.21, E09.22, E09.29, E09.311, E09.319, E09.3211, E09.3212, E09.3213, E09.3219, E09.3291 - E09.3293, E09.3299, E09.3311 - E09.3313, E09.3391 - E09.3393, E09.3399, E09.3411 - E09.3413, E09.3419, E09.3491 - E09.3493, E09.3499, E09.3511 - E09.3513, E09.3519, E09.3521 - E09.3523, E09.3529, E09.3531 - E09.3533, E09.3539, E09.3541 - E09.3543, E09.3549,  E09.3551 -  E09.3553,  E09.3559,  E09.3591 -  E09.3593,  E09.3599,  E09.36,  E09.37X1 - E09.37X3, E09.37X9, E09.39 - E09.44, E09.49, E09.51, E09.52, E09.59, E09.610, E09.618, E09.620 - E09.622, E09.628, E09.630, E09.638, E09.641, E09.649, E09.65, E09.69, E09.8, E09.9, E10.21, E10.22, E10.29, E10.311, E10.319, E10.3211 - E10.3213, E10.3119, E10.3291 - E10.3293, E10.3299, E10.3311 - E10.3313, E3319, E10.3391 - E10.3393, E10.3399, E10.3411 - E10.3413, E10.3419, E10.3491 - E10.3493, E10.3499, E10.3511 - E10.3513, E10.3519, E10.3521 - E10.3523, E10.3529, E10.3531 - E10.3533, E10.3539, E10.3541 -E10.3543, E10.3549, E10.3551 - E10.3553, E10.3559, E10.3591 - E10.3593, E10.3599, E10.36, E10.37X1 - E10.37X3, E10.37X9, E10.39 - E10.44, E10.49, E10.51, E10.52, E10.59, E10.610, E10.618, E10.620, E10.621,  E10.622, E10.628, E10.630, E10.641, E10.649, E10.65, E10.69, E10.8, E10.9, E11.00, E11.01, E11.10, E11.11, E11.21, E11.22, E11.29, E11.311, E11.319, E11.3211 - E11.3213, E11.3219, E11.3291 - E11.3293, E11.3299, E11.3311 - E11.3313, E11.3319, E11.3391 - E11.3393, E11.3399, E11.3411 - E11.3413, E11.3419, E11.3491 - E11.3493, E11.3499, E11.3511 - E11.3513, E11.3519, E11.3521 - E11.3523, E11.3529, E11.3531 - E11.3533, E11.3539, E11.3541 - E11.3543, E11.3549, E11.3551 - E11.3553, E11.3559, E11.3591 - E11.3593, E11.3599, E11.36, E11.37X1 - E11.37X3, E11.37X9, E11.39 - E11.44, E11.49, E11.51, E11.52, E11.59, E11.610, E11.618, E11.620 - E11.622, E11.628, E11.630, E11.638, E11.641, E11.649, E11.65, E11.69, E11.8, E11.9, E13.00, E13.01, E13.10, E13.11, E13.21, E13.22, E13.29, E13.311, E13.319, E13.3211 - E13.3213, E13.3219, E13.3291 - E13.3293, E13.3299, E13.3311 - E13.3313, E13.3319, E13.3391 - E13.3393, E13.3399, E13.3411 - E13.3413, E13.3419, E13.3491 - E13.3493, E13.3499, E13.3511 - E13.3513, E13.3519, E13.3531 - E13.3533, E13.3539, E13.3541 - E13.3543, E13.3549, E13.3551 - E13.3553, E13.3559, E13.3591 - E13.3593, E13.3559, E13.3591 - E13.3593, E13.3599, E13.36, E13.37X1 - E13.37X3, E13.37X9, E13.39 - E13.44, E13.49, E13.51, E13.52, E13.59, E13.610, E13.618, E13.620 - E13.622, E13.628, E13.630, E13.638, E13.641, E13.649, E13.65, E13.69, E13.8, E13.9, E23.2 |
| Urinary Tract Infection | A18.10, A18.11, A18.12, A18.13, A36.85, A52.75, A54.01, A56.11, A56.19, A98.5, B65.0, B90.1, N10, N11.0, N11.8, N11.9, N12, N13.5, N13.6, N15.1, N28.84, N28.85, N28.86, N30.00, N30.01, N30.10, N30.11, N30.20, N30.21, N30.30, N30.31, N30.80, N30.81, N30.90, N30.91, N34.0, N34.2, N34.3, N39.0 |

**Supplementary Table S4.** Charges From Hospital Billing Records Considered to be Urinary Catheter Placement

| **Charge Descriptions** |
| --- |
| TRAY CATHETER FOLEY W/REG BAG, ER INSERT TEMP INDWELLING BLADDER CATH SIMPLE, TRAY CATHETER FOLEY W/URINEMETER, INSERT TEMP INDWELLING BLADDER CATH SIMPLE, CATHETER FOLEY TEMP SENSING, CATHETER FOLEY, TRAY CATHETER URETHRAL, TRAY CATHETER FOLEY SILICONE W/REG BAG, CATHETER FOLEY COUDE, CATHETER FOLEY SILICONE, TRAY CATHETER URETERAL, CATHETER FOLEY 2W, TRAY CATHETER FOLEY ADDACATH, CATHETER URETHRAL, CATHETER URETERAL, CATHETER FOLEY 3W, TR INSERT TEMP INDWELLING BLADDER CATH SIMPLE, CATHETER URETHRAL 14FR, ER INSERT TEMP INDWELLING BLADDER CATH COMP, CATHETER URETERAL OPEN END, ER INSERTION NON-DWELLING BLADDER CATHETER, CATHETER FOLEY LATEX FREE, CATHETER FOLEY SPECIALTY, INSERT TEMP INDWELLING BLADDER CATH COMP, CATHETER URETERAL POLLACK, CATHETER URETHRAL 20FR, CATHETER FOLEY PEDIATRIC, CATHETER FOLEY COUNCIL, CATHETER URETHRAL 8FR, CATHETER URETHRAL 12FR, CATHETER URETERAL CONE TIP, CATHETER URETERAL FLEXIBLE, TR INSERT TEMP INDWELLING BLADDER CATH COMP, CATHETER URETERAL WHISTLE TIP, CATHETER URETHRAL 5FR, CATHETER ROBINSON/ROBNEL, CATHETER URETERAL WHISTLE TIP 5FR, PF INSERT TEMP INDWELLING BLADDER CATH SIMPLE, CATHETER URETERAL CONE TIP 8FR, CATHETER FOLEY SILICONE 3W, CATHETER URETERAL OLIVE TIP, CATHETER URETHRAL 10FR, CATHETER URETERAL WHISTLE TIP 4FR, CATHETER URETERAL BALLOON, PF INSERT TEMP INDWELLING BLADDER CATH COMP, CL INSERT TEMP INDWELLING BLADDER CATH SIMPLE, CATHETER RED RUBBER, CATHETER URETERAL SPIRAL TIP, *CATHETER FOLEY 12FR 5CC 2W, *CATHETER FOLEY 16FR 30CC 2W, *CATHETER FOLEY COUDE 16FR 2W 5CC, *CATHETER FOLEY COUDE 2W 5CC, ADAPTER CATHETER URO, CATHETER URETHRAL 4FR, *CATHETER FOLEY 14FR 5CC 2W, *CATHETER FOLEY 16FR 30CC 2W, *CATHETER FOLEY 20FR 30CC 2W, *CATHETER FOLEY 20FR 5CC, *CATHETER FOLEY 22FR 30CC 2W, *CATHETER FOLEY 22FR 30CC 3W, *CATHETER FOLEY 24FR 5CC 2W, *CATHETER FOLEY COUDE 2W 30CC, CATHETER URETERAL LIGHTED, CATHETER URETERAL OLIVE TIP 5FR, RN FOLEY CATHETER INSERTION |

**Supplementary Table S5.** Facility-Level Variability in Median Percent of Urine Cultures in Each Urinalysis Category

| **Characteristics** | **%Urine Culture with Positive Urinalysis** Median (Q1:Q3) | **%Urine Culture with Negative Urinalysis** Median (Q1:Q3) | **%Urine Culture without Urinalysis** Median (Q1:Q3) |
| --- | --- | --- | --- |
| **Overall** | 75.0 (55.6:86.4) | 15.0 (6.6:28.6) | 5.3 (2.2:10.5) |
| **Year** |  |  |  |
| 2017 | 71.4 (50.3:86.1) | 14.1 (5.4:31.3) | 6.3 (2.6:12.4) |
| 2018 | 75.9 (55.6:87.1) | 14.4 (5.9:28.6) | 5.5 (2.3:10.2) |
| 2019 | 75.1 (55.7:85.9) | 15.0 (6.5:27.3) | 5.4 (2.3:11.3) |
| 2020 | 76.7 (59.8:86.3) | 15.9 (7.9:27.3) | 4.5 (1.4:9.1) |
| **Geographic location** |  |  |  |
| Urban | 75.3 (55.6:86.5) | 14.3 (6.3:27.1) | 5.6 (3.2:10.5) |
| Rural | 76.9 (56.5:87.2) | 16.7 (7.6:31.3) | 4.4 (0.0:10.2) |
| **Teaching hospital** |  |  |  |
| Yes | 69.6 (53.7:83.9) | 20.0 (8.3:32.3) | 6.3 (3.2:10.5) |
| No | 76.9 (56.5:87.2) | 14.1 (5.9:26.2) | 4.9 (1.8:10.6) |
| **Number of beds** |  |  |  |
| <100 | 78.6 (62.5:88.5) | 15.0 (6.0:26.9) | 3.3 (0.0:9.1) |
| 100-199 | 76.6 (57.9:87.1) | 13.0 (5.3:26.1) | 4.6 (1.8:10.3) |
| 200-299 | 68.1 (45.3:82.5) | 16.9 (7.3:33.7) | 6.7 (3.3:12.7) |
| 300-399 | 75.1 (54.2:85.7) | 16.2 (7.2:29.9) | 5.1 (2.7:8.5) |
| 400-499 | 67.5 (49.2:82.7) | 18.9 (8.2:32.6) | 7.3 (4.3:13.2) |
| >499 | 74.2 (56.7:86.6) | 13.9 (7.1:26.9) | 6.9 (4.1:11.5) |
| **Facility region** |  |  |  |
| South | 75.9 (58.3:86.6) | 14.7 (6.6:25.0) | 4.8 (1.8:10.2) |
| Midwest | 77.8 (52:87.9) | 13.4 (5.1:33.2) | 6.3 (2.9:11.1) |
| Northeast | 62.6 (43.4:81.8) | 24.7 (9.3:42.9) | 6.3 (2.7:10.8) |
| West | 76.0 (49.4:83.9) | 14.9 (8.5:25.0) | 4.8 (1.4:11.1) |

* Data source was a dynamic cohort of U.S. acute care hospitals, participating in the PINC AI™ Healthcare Database (PHD).

**Supplementary Figure S1.** Variability in Hospitals’ Median Percentages of Urine Cultures (UCs) for category: UC With Positive Urinalysis, UC with Negative Urinalysis, and UC With No Urinalysis, 2017-2020 

Note: the horizontal lines represent the 1st quartile, median, and the 3rd quartile of the percent of all urine cultures in each group for all months for a hospital (n=277). The figure was created using R 3.6.1.

* Data source was a dynamic cohort of U.S. acute care hospitals, participating in the PINC AI™ Healthcare Database (PHD).
